# A novel green biosynthesis approach and structural characterization of Ag–Fe bimetallic nanoparticles using the red alga *Galaxaura rugosa*

**DOI:** 10.1038/s41598-025-16009-1

**Published:** 2025-08-24

**Authors:** Elham M. Ali, Ashraf Elsayed, Ahlam S. El Shehawy

**Affiliations:** 1 Department of Environmental Studies, The National Authority for Remote Sensing and Space Sciences (NARSS), Cairo, Egypt; 2 Department of Aquatic Environment, Faculty of Fish Resources, University of Suez, Suez, Egypt; 3 Department of Botany, Faculty of Sciences, University of Mansoura, Mansoura, Egypt

**Keywords:** Algae, *Galaxaura rugosa*, Biosynthesis, Ag–Fe bimetallic nanoparticles, Characterization, Biotechnology, Environmental sciences, Nanoscience and technology

## Abstract

A novel green and eco-friendly approach has been used to biosynthesize Ag–Fe bimetallic nanoparticles (Ag–FeBNPs) by using the water extract of the red alga species; *Galaxaura rugosa.* The surface plasmon resonance band of Ag–FeBNPs is positioned at 327 nm. X-ray diffraction analysis (XRD) illustrated the crystalline nature of biogenic nanoparticles with average diameters of 32.6 nm. Transmission electron microscopy (TEM) and selected area electron diffraction (SAED) showed that the particles have a crystalline spherical shape with a size range from 19.95 to 37.11 nm. Scanning electron microscopy (SEM) and Energy dispersive analysis (EDAX) give the surface morphology and elemental composition of Ag–FeBNPs, which are spherical in high intensity. Fourier transmittance infrared spectroscopy (FTIR) showed various stretching vibrations at 3421, 1598, 1384, 1035, and 865 cm^−1^. These findings suggest that biomolecules play a crucial role in forming and stabilizing Ag–FeBNPs. Zeta potential values show − 16.1 mV. This study demonstrates the promising future of Ag–FeBNPs for nanobiotechnology and nanoscience, offering an environmentally friendly and simple approach for nanoparticles biosynthesizing. In addition, the synthesized Ag–FeBNPs exhibit properties that make them suitable for potential applications in biomedical fields, environmental remediation, and catalysis.

## Introduction

Nanotechnology is one of the top technologies that occurs at the nanoscale (1 × 10^−9^), where nanomaterials behave differently from their bulkier counterparts^1^. Nanoparticles (NPs) fabrication methods can be largely divided into physical methods, chemical methods, and biological methods^2^. Nowadays, researchers have concentrated on using a biological approach to synthesize NPs since it is typically less expensive, nontoxic, scalable, and harmless to the environment^3–5^. Because of their ease of culture and handling, low energy requirement, reduced toxicity, and low environmental risk, algae are therefore being investigated extensively as a potential tool for the green synthesis of NPs^6^. Among all algae, the red algae stand out as the most diverse group, thriving in many tropical and subtropical intertidal communities that harbor a wealth of bioactive compounds, including polysaccharides, lipids, and polyphenols^7^. Ranging from single-celled forms to intricate multicellular structures, they represent a vital component of aquatic photoautotrophic plant life^8^. *Galaxaura rugosa* as a red marine algae is a rich source of bioactive compounds that could be used as reducing and capping agents for phyco-synthesis of NPs^9^. Our previous study reported the feasibility of using *G.rugosa* alga to synthesize silver NPs^10^.

Compared to their bulk and monometallic counterparts, bimetallic nanoparticles have improved optical, electronic, magnetic, and catalytic characteristics^11,12^. Silver NPs are characterized by chemical stability, flexibility, biological activity, strong surface plasmon resonance (SPR) absorption, and high electron capture performance in the visible region^13^. Iron NPs possess many exceptional properties like low toxicity, low cost, and magnetic properties^14^. When silver and iron are combined into bimetallic nanoparticles, their synergistic interaction often results in enhanced multifunctional behavior that surpasses the capabilities of individual monometallic nanoparticles^15^. Specifically, Ag–FeBNPs exhibit both strong plasmonic effects and magnetic properties, which make them highly effective in a range of applications. The bimetallic structure allows for improved electron transfer, increased catalytic activity, and greater stability. These combined effects make Ag–FeBNPs more versatile and efficient than either Ag or Fe NPs alone^16^. It has been found that the optical, magnetic, and electronic properties of silver and iron NPs can be further varied when they are combined through a nanoscale interface to form heterodimeric NPs because the surface plasmon band’s extinction coefficient is significantly higher and one can provide silver characteristics a magnetic behavior, there has been a rise in interest in designing Ag–Fe bimetallic NPs (Ag–FeBNPs)^17^. The unique composition and structure of Ag–FeBNPs have sparked significant research interest due to their diverse applications across various fields, including biomedical applications^18^, environmental remediation^19^, sensor development^20^, and catalysis^21^.

Therefore, this study aims to investigate the phyco-synthesis of Ag–FeBNPs by the red alga *G. rugosa*. Furthermore, various characterization techniques were employed to gain a deeper understanding of the properties and characteristics of the phyco-synthesized Ag–FeBNPs. To the best of our knowledge, this is the first report on the green synthesis of Ag–FeBNPs using *G. rugosa*.

## Materials and methods

### Seaweed collection and extract preparation

Marine algae; namely *Galaxaura rugosa* were collected by hand from the littoral zone in June 2021 from Zaafarana beach (latitudes 29.06” N and longitudes 32.43” E) located in the Suez Gulf, the Red Sea, Egypt; it is 82 km south of Alain AL Sokhna. The algal extract was filtered through Whatman Filter paper No.1 and the provided supernatant was used to prepare the NPs. Algal extract was prepared by soaking 5 g of algal powder in 100 ml of deionized water for 24 h with shaking at 25 ± 2 °C (room temperature). The pH of the resulting extract was measured and found to be 6.8. The extract was filtered (Whatman No. 1) and the supernatant used for nanoparticle synthesis^22^.

### Phyco-synthesis of Ag–FeBNPs

Phyco-synthesis of Ag–FeBNPs was carried out by mixing solutions of AgNO_3_ (20 ml, 0.01 M) and FeCl_3_ (20 ml, 0.01 M) with constant magnetic stirring at 25 ± 2 °C for 20 min. Then, 20 ml of freshly prepared aqueous extract was added to the Ag–Fe solution under continuous stirring (with a measured pH of 6.8). The mixture was stirred for an additional 30 min at the same temperature. The reaction progress was monitored by observing the colour change, indicating the Ag–FeBNPs’ formation. The synthesized nanoparticles were centrifuged at 12,000 rpm for 20 min and then washed several times with deionized water for further characterization^23^. Zeta potential analysis was done to the nano-colloidal solution to test stability and surface charge of the phyco-synthesized Ag–FeBNPs using the Photon Correlation Spectroscopy (PCS) (Malvern Zeta size Nano-zs90, U.S.A), with deionized water as the dispersion medium at 25 °C, and an applied voltage of 150 V.

### Characterization of Ag–FeBNPs

The prepared Ag–FeBNPs were characterized by the UV–vis absorption spectra in the 200–600 nm wavelength range using UV-visible absorption spectroscopy (Unicam UV-VIS. Spectrometer UV2, U.S.A). XRD analysis was obtained by a DX-1000 X-ray powder diffractometer run at 40 kV and 30 mA, in the 2θ range of 10°–90°. The size and shape of the nano-colloidal sample were visualized by TEM (JEOLJEM-2100, U.S.A) using a carbon-coated grid (Type G 200, 3.05µ diameter, TAAP, U.S.A)^24^. The crystalline structure was described through the crystallographic experimental technique performed inside the TEM by SAED. ImageJ software (version 1.53, NIH, USA) was used to analyze TEM images and measure the particle size distribution for at least 100 nanoparticles to ensure statistical reliability. Also, the FEI-TITAN 80–300 kV SEM was used to study the morphological structure of the NPs, where a powder form of NPs was cast onto glass slides, followed by fixation on copper supports, then covered with a thin layer of gold by sputtering. The elements presented in NPs were determined by EDX integrated into the SEM^25^.

## Results and discussion

### Taxonomic description of the used seaweed

**Fig. 1:**
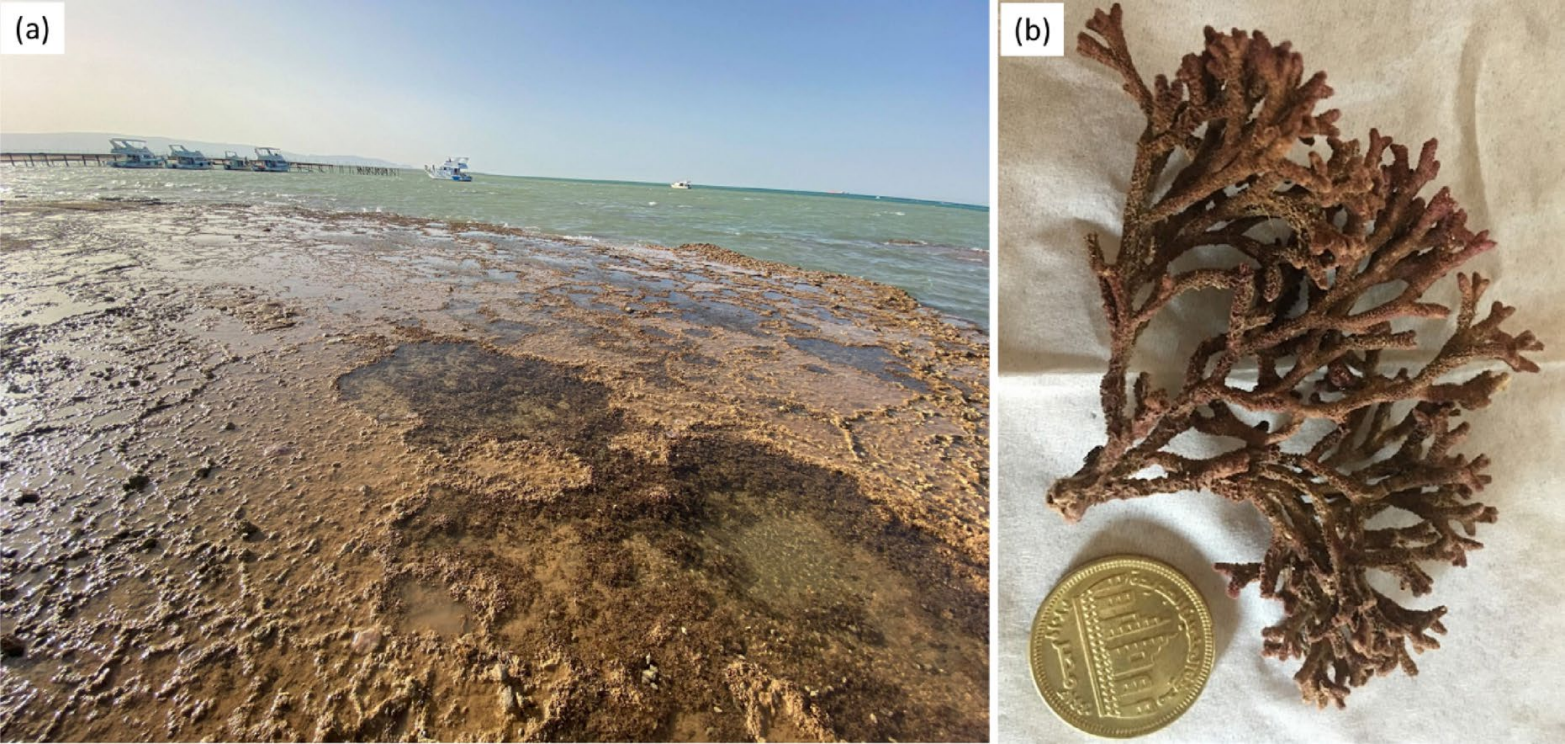
Photos representing **(a)** the study area and the collected seaweed **(b)** *Galaxaura rugosa.*

Species identification and taxonomical classification were done by a classification expert using fresh samples on the same collection day. Algae are identified as red algae (Rhodophyta) namely; *Galaxaura rugosa* (J.Ellis & Solander), family Galaxauraceae (Fig. 1). The collected alga species were classified according to the previous literature^26^.

### UV-Vis analysis of the phyco-synthesized Ag–FeBNPs

**Fig. 2:**
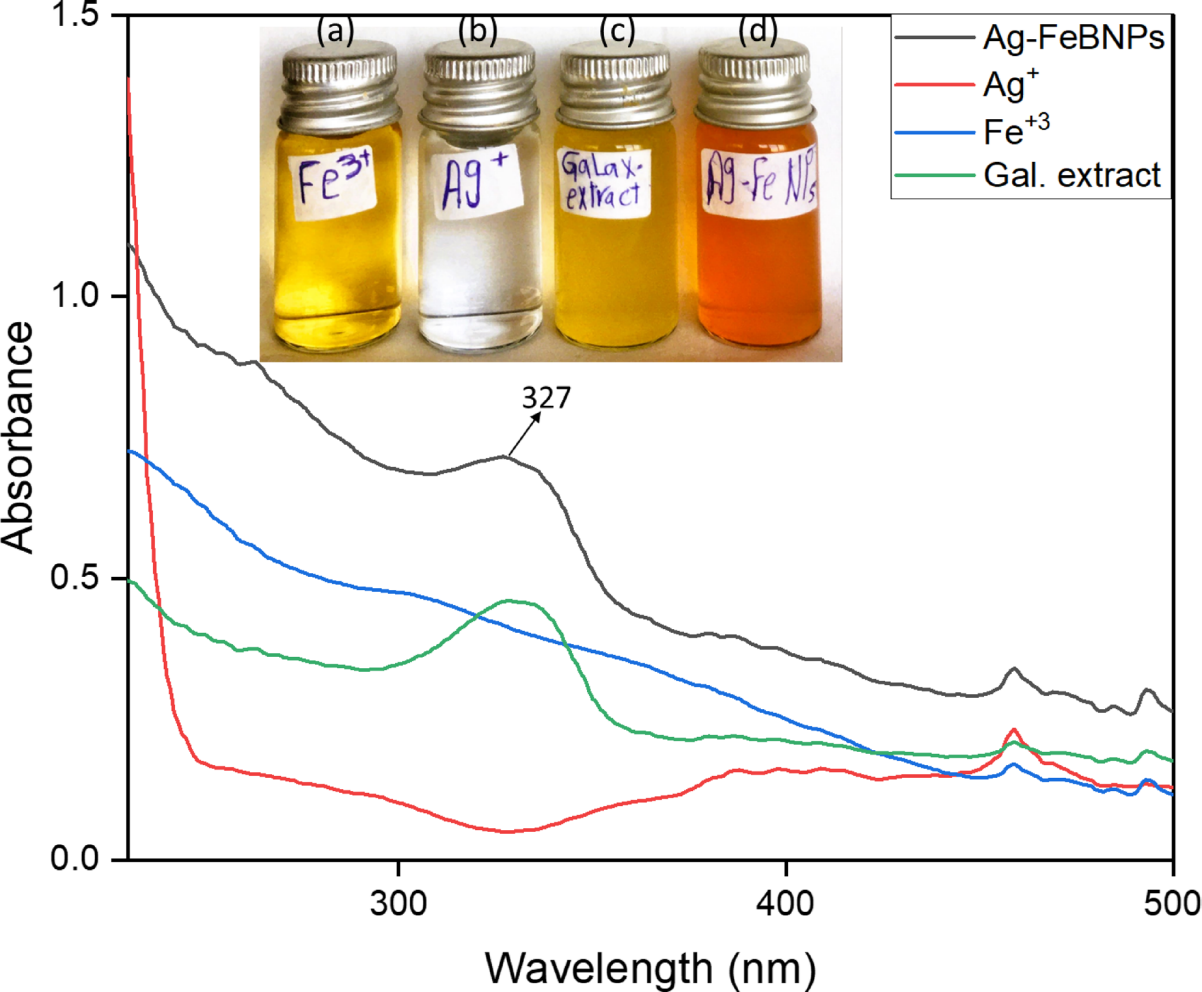
UV-vis spectra of the phyco-synthesized Ag–FeBNPs capped by *Galaxaura rugosa* marine algal extract, inset: shows (a) FeCl_3_, (b) AgNO_3_, (c) *G. rugosa* extract, and (d)Ag–FeBNPs.

The color variation of the algal extract was obvious on visual observation from yellow to yellowish brown. This transition of color is mainly attributed to the algal active compounds that act as reducing, stabilizing, and capping agents^27^. UV-visible spectroscopy showed the maximum absorption peak of Ag–FeBNPs at 327 nm, as shown in Fig. 2. This characteristic band can be attributed to the surface plasmon response produced by the interaction between the electrons in the conduction band on the surface of the NPs and the incoming light^28^. This observed absorption peak is consistent with previous studies on biologically synthesized Ag–Fe bimetallic nanoparticles, where surface plasmon resonance bands were reported between 320 and 340 nm, depending on the synthesis conditions and capping agents used^29,30^.

To further evaluate the optical behavior of the synthesized Ag–FeBNPs, the optical band gap energy (Eg) was estimated using the Tauc plot method. The Tauc plot derived from UV–Vis data (plotting (αhν)² vs. photon energy hν) revealed an estimated band gap of approximately 2.85 eV, indicating the semiconducting nature of the nanoparticles. This band gap value suggests potential applicability in photocatalytic and optoelectronic applications and aligns with reported values for Ag–FeBNPs synthesized via green routes^31^.

### FTIR spectroscopic analysis and XRD analysis of the phyco-synthesized Ag–FeBNPs

**Fig. 3:**
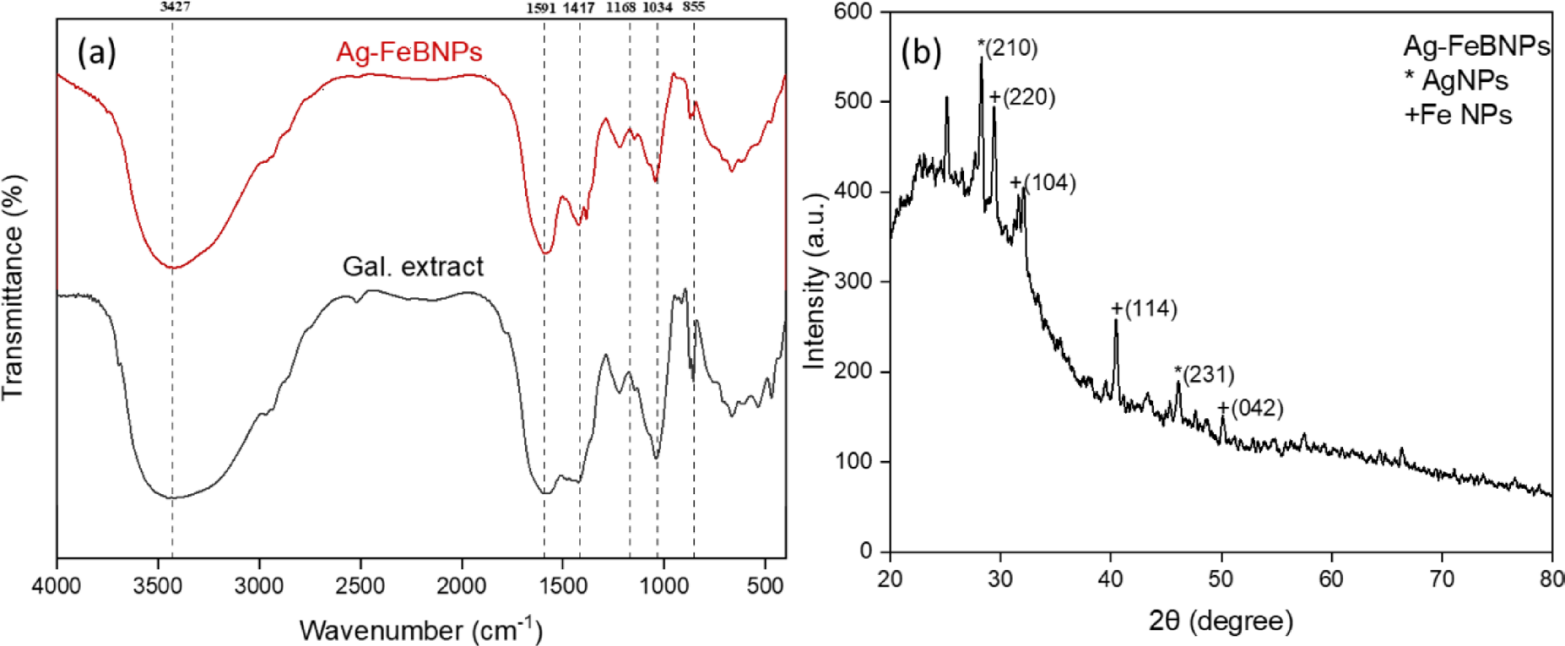
(**a**) FTIR of the phyco-synthesized Ag–FeBNPs and *G. rugosa* marine algal extract; (**b**) XRD pattern of Ag–FeBNPs capped by *G. rugosa* marine algal extract.

The FTIR spectrum of the aqueous extract of *G. rugosa* (Fig. 3a) exhibits a distinctive peak at 3421 cm^−1^, attributed to the O-H stretching vibrations within polyphenols and OH groups of sugar rings^32^. The absorption peaks that appeared at 1598 cm^−1^ may be due to the vibrations in the C = O bond stretching within the aromatic rings of different phenolic compounds, including polyphenols and flavonoids that are present in extracts of both algal species^33^. The spectrum of peaks determined at 1384 cm^−1^ represents the N = O bond (nitro groups), at 1035 cm^−1^ represents the C-O stretching of primary alcohols, at 672 cm^−1^ represents aromatic rings of the C-H bond, and that at 865 cm^−1^ represents the C-H stretching (vinyl groups)^34^. The phyco-synthesized Ag–FeBNPs possess various stretching vibrations, as depicted in Fig. 3a. Major peaks appeared at 3421, 1598, 1384, 1035, and 865 cm^−1^, in correspondence to the stretching of the N-H bond in amines, and O-H in alcohols, C = C bond stretching in alkene, N-O bond stretching in the nitro compound, O-H bond stretching in carboxylic acid, and C = C bond bending in alkenes, respectively. The Ag–FeBNPs showed a minor shift with slight changes, indicating that the main biomolecules present in extracts were capped to the NPs surface as reported by El-Kassas and El Komi^35^. These shifts confirm that functional groups from bioactive compounds were actively involved in stabilizing the nanoparticles by binding to their surface, forming a protective layer that prevents agglomeration and improves colloidal stability^36^.

These biomolecules-particularly phenolic compounds, flavonoids, and hydroxyl-rich metabolites-likely acted as dual-function agents: reducing both Ag⁺ and Fe³⁺ ions into their respective metallic states and stabilizing them via capping. The simultaneous reduction of both metal ions nearby may promote the formation of bimetallic nanoparticles either as core-shell structures or alloyed forms, depending on kinetic and thermodynamic factors. The functional groups (-OH, -COOH, -NH₂) facilitate nucleation and prevent agglomeration, enabling the successful integration of Ag and Fe atoms into a single bimetallic nanostructure^29^.

A comparative looks at other algae-based nanoparticle syntheses further supports these findings. For instance, *Sargassum wightii* and *Ulva lactuca* extracts have demonstrated similar FTIR peaks associated with hydroxyl and carbonyl groups that contribute to both metal ion reduction and stabilization^36,37^. These similarities affirm the generalizable role of algal biomolecules in green nanoparticle synthesis across species. These results are in good agreement with several previous reports^38,39^.

The crystal structure of Ag–FeBNPs is clearly evident in Fig. 3b. The XRD pattern of Ag–FeBNPs from *G. rugosa* give the diffraction peaks at 2θ values of 28.25°, 29.34°, 31.57°, 40.49°, 46.31°, and 50.31° corresponding to the crystalline planes (210), (220), (104), (114), (231), and (042), emphasizing the face-centered cubic phase of the phyco-synthesized NPs. The diffraction peaks observed in the XRD pattern of Ag–FeBNPs are composed of the standard peaks of Ag (JCPDS no. 04-0783) and Fe (JCPDS no. 33–0664), demonstrating the formation of bimetallic phases of Ag and Fe^27^.

### Morphological analysis of Ag–FeBNPs

**Fig. 4:**
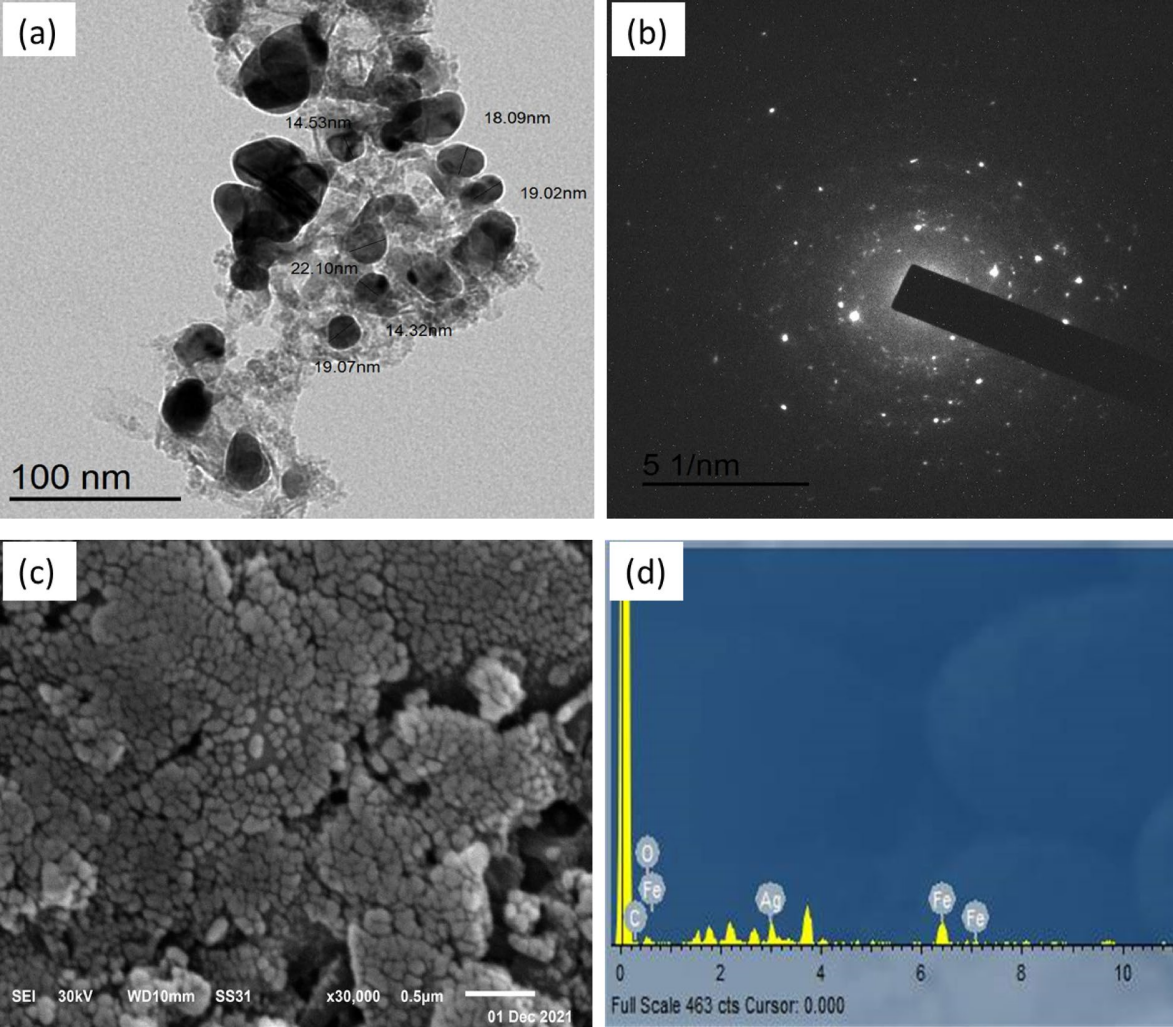
**(a)** TEM, **(b)** SAED, **(c)** SEM, and **(d)** EDX of the phyco-synthesized Ag–FeBNPs capped by *G. rugosa* marine algal extract.

The TEM micrograph of Ag–FeBNPs for *G. rugosa* shows an agglomeration of nano-spheres with particle sizes from 19.95 to 37.11 nm (Fig. 4a) as reported by Kamli, et al.^39^ who demonstrated the production of spherical Ag–Fe nanoparticles within a range of 100 nm using Beta vulgaris extract. Figure 4b shows the SAED pattern, which exhibits alternating dots and concentric rings, demonstrating that these NPs are polycrystalline^40^.

SEM analyses showed that the particle shapes were spherically shaped, highly distributed, and polydisperse with uniform surfaces (Fig. 4c). These results are in reasonable agreement with previous reports^39^. The chemical composition of Ag–FeBNPs was securitized upon EDX analysis, as depicted in Fig. 4d. The Fe and Ag signals shown in the EDX spectrum confirmed that these NPs are composed of AgNPs and FeNPs^27^. Ag–FeBNPs were composed of Fe with 13.51% by weight and 4.31% by atomic percentage, while Ag constituted 14.09% by weight and 2.33% by atomic percentage. These values provide a more precise quantitative assessment of the elemental composition, supporting the successful integration of both metals into the bimetallic structure. The existence of some extra peaks in the spectrum could be attributed to the participation of algal biomolecules in the NPs’ synthesis^41^.

### Zeta potential analysis

**Fig. 5:**
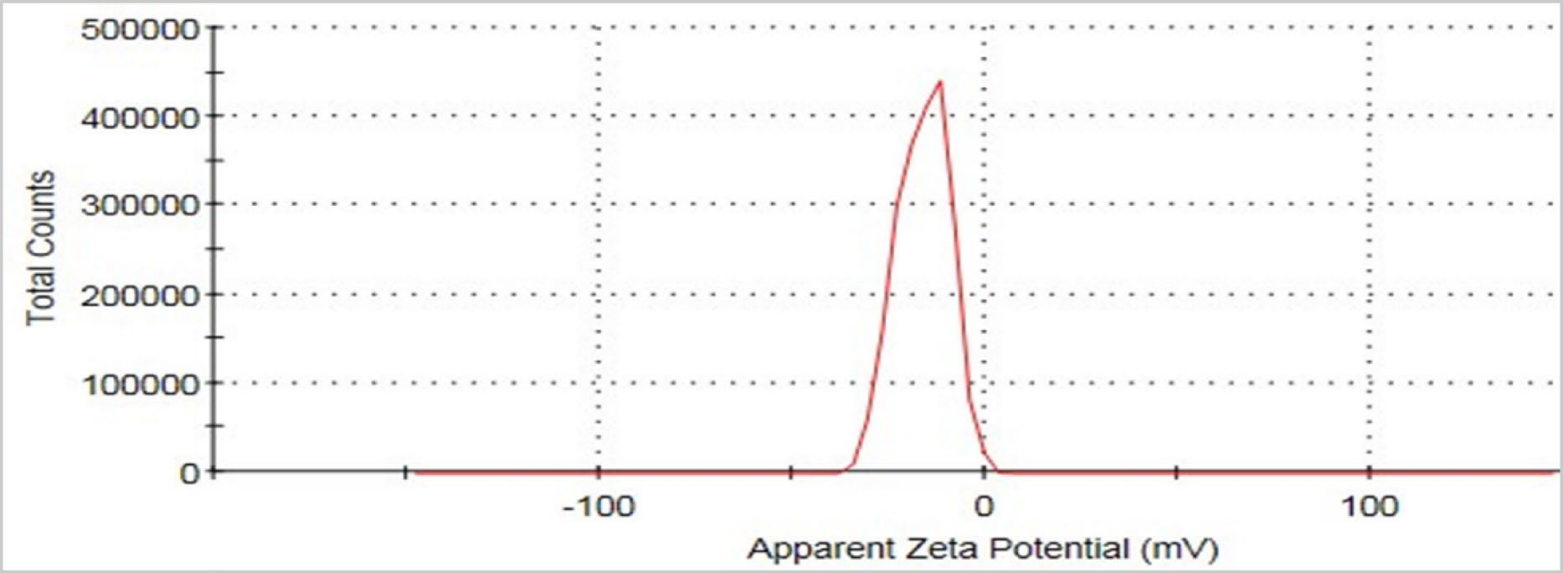
Zeta potential of Ag–FeBNPs phyco-synthesized from *G. rugosa* extract.

Zeta potential, which refers to the degree of stability of the colloidal particles, was used to determine the degree of electrostatic repulsion between adjacent similarly charged particles. The Ag–FeBNPs show negative potential values of 16.1 mV (Fig. 5). This coincides well with previous studies^42^. These negative potential values could be due to the capping impact of the biomolecules present in the algal extracts^43^. A zeta potential value of −16.1 mV indicates moderate colloidal stability, suggesting that while some electrostatic repulsion is present, aggregation may still occur over time, especially under varying pH or ionic strength conditions. This implies that the nanoparticles may remain sufficiently stable for short-term applications but might require further stabilization for long-term storage or industrial use^44^.

This level of zeta potential suggests that while the nanoparticles are dispersible, their long-term colloidal integrity could be compromised under stress conditions, such as high salinity or variable temperatures, which may limit certain industrial applications without further surface modification^45^. Although this study focused on nanoparticle synthesis and characterization, long-term stability tests and functional performance evaluations (e.g., antimicrobial activity, catalytic efficiency, or adsorption capacity) were not within the scope and are planned for future work.

A comparative analysis with previously reported biosynthesized Ag–FeBNPs using different biological sources, such as fungi and plants, is presented in Table 1. The particle size of Ag–FeBNPs in our study (19.9–37.1 nm) falls within the smaller range compared to fungal-derived nanoparticles, such as those synthesized using *Gymnascella dankaliensis* (~ 96.8 nm)^29^. While *Gardenia jasminoides*-derived Ag–FeBNPs exhibited the smallest size (~ 13 ± 6.3 nm) and core–shell morphology, our nanoparticles displayed a spherical shape and moderate zeta potential of −16.1 mV, indicating acceptable colloidal stability for short-term applications.

Compared to *Beta vulgaris*-mediated Ag–FeBNPs (~ 15 nm), which showed antifungal and apoptosis-inducing activities^39^, the *G. rugosa*–derived nanoparticles remain primarily characterized, with functional applications such as antimicrobial or catalytic assessments planned for future work. Additionally, while *Gardenia jasminoides*-synthesized Ag–FeBNPs demonstrated magnetic behavior and broad-spectrum antimicrobial potential^46^, our synthesis emphasizes eco-friendliness and ease of preparation. These comparisons highlight the uniqueness of using red marine algae as a sustainable and efficient route for bimetallic nanoparticle biosynthesis.

**Table 1:** Comparison of biologically synthesized Ag–Fe bimetallic nanoparticles using different green sources.

Biological Source	Size (nm)	Shape	Zeta Potential (mV)	Stability/Applications	Reference
*Galaxaura rugosa* (Red alga)	19.9–37.1	Spherical	−16.1	Moderate colloidal stability; synthesis and characterization	Current study
*Gymnascella dankaliensis* (Fungus)	~ 96.8	Spherical	Not reported	Antibacterial, anticancer, dye removal, seed germination	^29^
*Gardenia jasminoides* (Plant)	~ 13 ± 6.3	Core-shell	−17 to −21	Magnetic behavior; broad-spectrum antimicrobial activity	^15^
*Beta vulgaris* (Plant)	~ 15	Spherical	Not reported	Antifungal; ROS-mediated apoptosis in Candida auris	^39^

## Conclusions

The phyco-synthesis of Ag–FeBNPs emerges as a sustainable and eco-friendly approach for green nanotechnology. This study demonstrates the promising potential of the red algae *Galaxaura rugosa* in this process. Using *G. rugosa* extract, we successfully produced Ag–FeBNPs with well-characterized properties, as confirmed by various analytical techniques (UV-vis, FTIR, XRD, TEM, SAED, SEM, EDAX, zeta potential). The richness of bioactive molecules within *G. rugosa* suggests a cost-effective route for large-scale nanoparticle production through green methods. These phyco-synthesized Ag–FeBNPs exhibit unique properties that warrant further exploration in diverse fields, including environmental remediation, biomedicine, and catalysis. Further studies are needed to fully understand their potential benefits and limitations. This study undoubtedly paves the way for exciting advancements in the utilization of phyco-synthesized Ag–FeBNPs.

## Data Availability

The data will be provided by the corresponding author upon reasonable request.
